# Individuals departing non-breeding areas early achieve earlier breeding and higher breeding success

**DOI:** 10.1038/s41598-024-53575-2

**Published:** 2024-02-19

**Authors:** Fraser Bell, Janne Ouwehand, Christiaan Both, Martins Briedis, Simeon Lisovski, Xuelai Wang, Stuart Bearhop, Malcolm Burgess

**Affiliations:** 1 Centre for Ecology and Conservation, University of Exeter, Penryn, Cornwall UK; 2 Royal Society for the Protection of Birds, Centre for Conservation Science, The Lodge, Sandy, Bedfordshire UK; 3 Conservation Ecology Group, University of Groningen, Groningen, The Netherlands; 4 Department of Bird Migration, Swiss Ornithological Institute, Sempach, Switzerland; 5 Lab of Ornithology, Institute of Biology, University of Latvia, Rīga, Latvia; 6 Alfred Wegener Institute for Polar and Marine Research, Telegrafenberg, Potsdam, Germany; 7 Centre for Research in Animal Behaviour, University of Exeter, Exeter, Devon UK; 8 PiedFly.Net, Yarner Wood, Bovey Tracey, Devon, UK

**Keywords:** Animal migration, Behavioural ecology

## Abstract

Conditions experienced by an individual during migration have the potential to shape migratory tactic and in turn fitness. For large birds, environmental conditions encountered during migration have been linked with survival and subsequent reproductive output, but this is less known for smaller birds, hindering our understanding of mechanisms driving population change. By combining breeding and tracking data from 62 pied flycatchers (*Ficedula hypoleuca*) representing two breeding populations collected over 2016-2020, we determine how variation in migration phenology and tactic among individuals affects subsequent breeding. Departure date from West African non-breeding areas to European breeding grounds was highly variable among individuals and had a strong influence on migration tactic. Early departing individuals had longer spring migrations which included longer staging duration yet arrived at breeding sites and initiated breeding earlier than later departing individuals. Individuals with longer duration spring migrations and early arrival at breeding sites had larger clutches, and for males higher fledging success. We suggest that for pied flycatchers, individual carry-over effects may act through departure phenology from West Africa, and the associated spring migration duration, to influence reproduction. While our results confirm that departure date from non-breeding areas can be associated with breeding success in migratory passerines, we identify spring staging duration as a key component of this process.

## Introduction

Migratory species have complex lifecycles with multiple distinct periods spent in different locations and environments that are connected across the annual cycle^1^. As such, the events and processes experienced by individuals during one season can have fitness repercussions as carry-over effects at subsequent stages^2^. For migrants, these can arise due to variation in the extent to which individuals access or use resources at non-breeding areas^3,4^, or how they respond to environmental conditions experienced during migration^5,6^, which in turn act on migratory behaviours and timing.

Carry-over effects can be important mechanisms driving variation in migratory bird breeding success^2^, especially for long-distance migrants that experience variable environments and conditions within a single seasonal movement. Annual variation in climate at non-breeding or staging areas, or weather encountered during migration, can determine the timing and duration of spring migration with implications for breeding phenology, clutch size, and fledging success^5,7,8^. Similarly, occupancy of low-quality sites^9^ or habitats^3,10^ during the non-breeding period can delay the onset of spring migration, with individuals consequently more likely to arrive at breeding sites in poorer physical condition, which can delay breeding^11,12^. Refuelling rates or duration of stops at staging areas can also influence migratory timings, fuel load^13^, breeding success^14^, and annual survival^15^.

For songbirds, links have been made between environmental conditions experienced within the non-breeding season and subsequent breeding timing^16,17^ and success^18^. Tracking studies suggest that in response to environmental conditions, individuals can flexibly adjust the timing of spring migration^19,20^ and duration of spring staging^4,21^, which can promote earlier arrival to breeding sites with associated breeding benefits such as improved fecundity^10^. However, it is less well known how the initiation of spring migration from non-breeding areas influences spring staging behaviours, for example how departure timing can affect the timing and duration of stops, and reproductive success in the subsequent breeding season.

Migratory birds have trade-offs between migratory timing and duration with survival and fitness; migration is energetically costly and associated with risks to survival^22^, while the pre-breeding spring migration is under strong selection for early arrival to breeding sites^23^ but potentially countered by fitness costs related to early arrival, such as reduced survival^24^. We have a limited understanding of where in the annual cycle migratory timing ‘decisions’ are made and whether individuals strategically speed up or slow down migratory movements to accommodate delays or adverse conditions. Migrating individuals may depart non-breeding areas earlier, but migrate slower and stop more often, a tactic associated with breeding benefits, in for example, horned larks *Eremophila alpestris* which had higher nest success and produced more fledglings^14^. Alternatively, individuals can remain in non-breeding areas for longer but compensate for delayed departure by migrating faster^4,25^, though this may incur survival costs^26^. These strategies can be mediated by environmental conditions however, as individuals seek to undertake migratory flight with supportive weather^27^.

A further consideration is that sex-differentiated spring migration patterns are well known in songbirds^28^, males often commence spring migration earlier and use staging areas earlier^14^, with females remaining in non-breeding areas or at staging areas for longer^29^. Consequently, males and females can experience different conditions during migration, yet the potential effects of this are poorly understood^30^. This is important as carry-over effects can be sex-specific^10,29^, for example male house martins *Delichon urbicum* that used wetter habitats during the non-breeding season produced more offspring in the following breeding period compared to males in drier habitats, which was not found for females^31^. As males benefit from early arrival to breeding sites in securing a territory and improving pairing opportunity^12,32^, delayed initiation of spring migration, or prolonged staging behaviour, may be associated with reduced male breeding success. Breeding related benefits for females are often linked to arrival in good physiological condition promoting investment in breeding^33^, as such, an increased staging duration and the associated increase of refuelling time may be associated with increased investment in breeding^34^.

Here we investigate how male and female pre-breeding spring migration departure date and migratory tactic interact with arrival date to breeding grounds, and interactions between these and breeding performance. We combined tracking and breeding data for individuals from two breeding populations of pied flycatcher *Ficedula hypoleuca*. Pied flycatchers from these populations show a high within-population and between-sex variation in departure date from West African non-breeding areas, in spring migration duration and in arrival date to breeding areas^35–37^. Within-individual variation is lower, with arrival dates to breeding grounds repeatable in both males and females, in terms of rank order of arrival^38^. Although Nicolau et al.^36^ showed no relationship between arrival date and clutch size at the population level, how such timing variation influences subsequent reproduction is largely unknown. For pied flycatchers, climate in West African non-breeding areas and the weather experienced during spring migration can influence the timing of arrival to breeding sites, timing and investment in breeding and breeding success at the population level^39,40^. Considering that population regulation of British breeding pied flycatchers appears primarily caused by factors operating outside the breeding period and hence potentially on migration^41^, like for many other migratory birds^42^, we aim to identify how events that cause differences in migratory timings and tactics during the spring migration can influence subsequent breeding.

## Results

In total 82 individuals fitted with geolocators were recaptured the breeding season after deployment (Supplementary Table S1), 44 from East Dartmoor (31 male, 13 female), and 38 from Drenthe (32 male, 6 female). 10 recovered geolocators were not analysed (where tags failed to record data, or the data were not useable, likely due to shading from feathers following moult), with data for 72 individuals available in total. Since batteries failed variably over the course of the annual cycle for 15 tags we derived incomplete migration cycles. Departure date from non-breeding areas was estimated for 62 individuals, and complete spring migrations (to breeding sites) for 57 individuals, 15 females and 42 males (Supplementary Fig. S1). Previous tag assessment from the same study populations showed no effects from tagging pied flycatchers on their behaviour, breeding success, migratory phenology, or apparent annual survival^43,44^.

### Departure date from non-breeding area

There was substantial variation in departure dates with the earliest departing 09 March and the latest 26 April. Males departed on average 5 days earlier than females (average male departure 27 March, range 08 March to 14 April, females 01 April, range 09 March to 25 April). Weather predictors within a 30-day period prior to departure did not influence the night-to-night likelihood of departure (*n* = 62, Supplementary Table S2). Fixed effects of year, breeding population (UK or Netherlands) and longitude of non-breeding location in West Africa had no impact on the likelihood of departure during a certain night. A second model without the inclusion of year to account for the possibility of between-year variation in response to certain weather conditions yielded similar results (Supplementary Table S2).

### Spring migration duration

**Figure 1:**
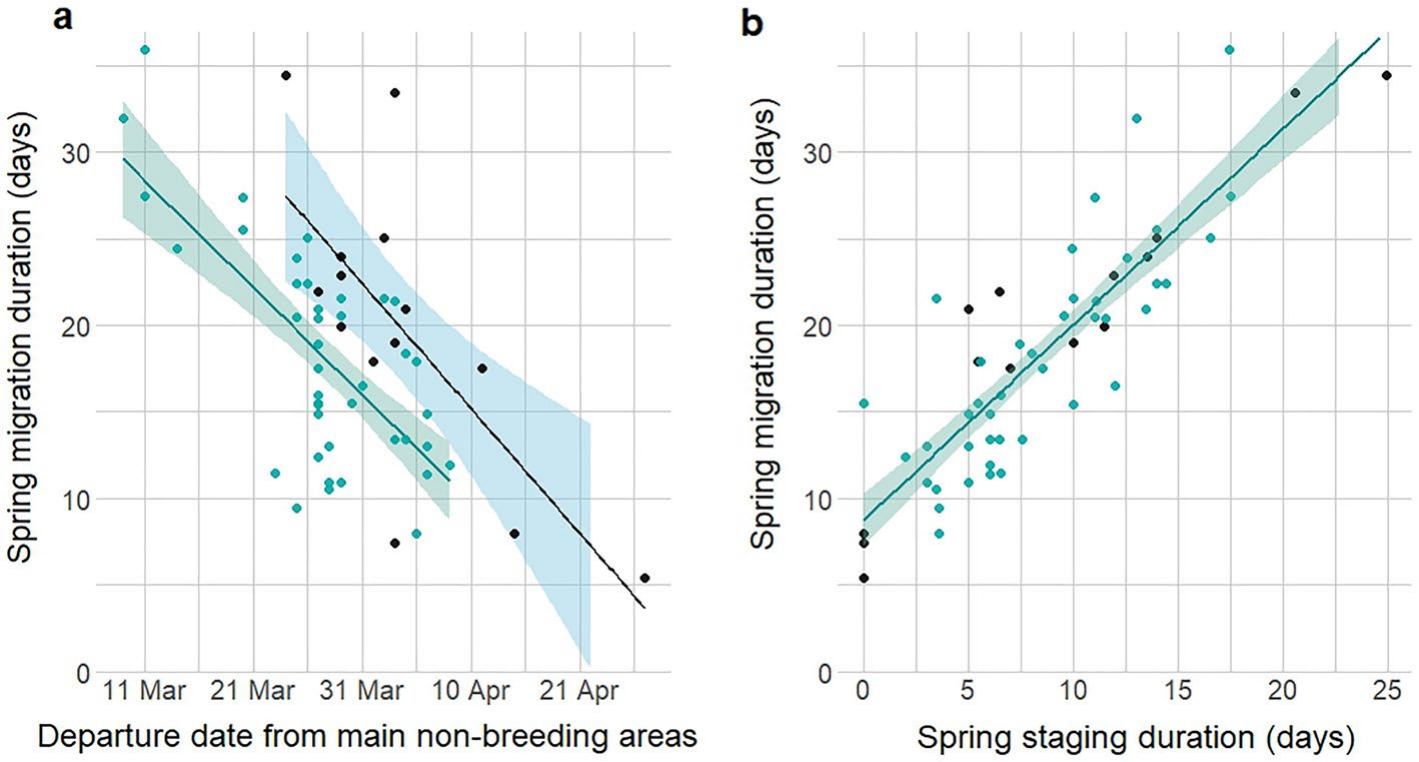
The relationships between spring migration duration and non-breeding departure date from African non-breeding grounds (**a**), and spring staging duration (**b**), in pied flycatchers tracked with geolocators. Females (14) are shown in black, males (42) green, (**a**) has a separate line for each sex while a single line is shown for all individuals combined in (**b**). Duration is displayed in days, and shading indicates 90% confidence intervals. Spring staging duration was defined as the total number of days at locations with stationary periods of >=2days, with a staging duration of 0 for individuals that did not stop.

There was a negative effect of departure date on duration of migration, with individuals departing non-breeding areas earlier having longer duration spring migrations. Spring migration duration varied across individuals (mean = 19 days; range = 4–59 days; n = 56, Fig. 1a and Table 1, all model sets shown in Supplementary Table S3), and individuals with longer migrations had longer staging duration (spring staging duration defined as a stationary period of >=2 days, Fig. 1b). Accounting for sex differences in departure date, there was an effect of sex on migration duration, with females migrating for a longer duration (Females: mean = 20 days; range = 6–34 days; n = 14; Males: mean = 18 days; range = 8–36 days; n =42), and stopping for longer on spring migration (Females: mean = 9 days; range = 0–25 days; n = 14; Males: mean = 8 days; range = 0–18 days; n =42).

**Table 1:** Candidate models testing an association between departure date from non-breeding areas and the spring migration duration.

(a) Component	Log Likelihood	AICc	ΔAIC	Weight
Departure date + Sex	**− 167.78**	**344.3**	**0**	**0.73**
Departure date + Sex + Population	− 167.71	346.6	2.29	0.23

(b) Fixed effects	Estimate ± SE	95% CI	*t* value	*P* value
Departure from non-breeding areas	− 0.65 ± 0.09	− 0.83/−0.47	− 7.18	< 0.001
Females	− 6.28 ± 1.66	− 9.53/− 3.03	− 3.39	< 0.001

All models within 4 ΔAIC of the top model are shown, with the best supported model in bold (a). Parameter estimates, adjusted standard error (SE), 95% confidence intervals (CI), and significance values from the best supported model (b).

### Arrival phenology

**Figure 2:**
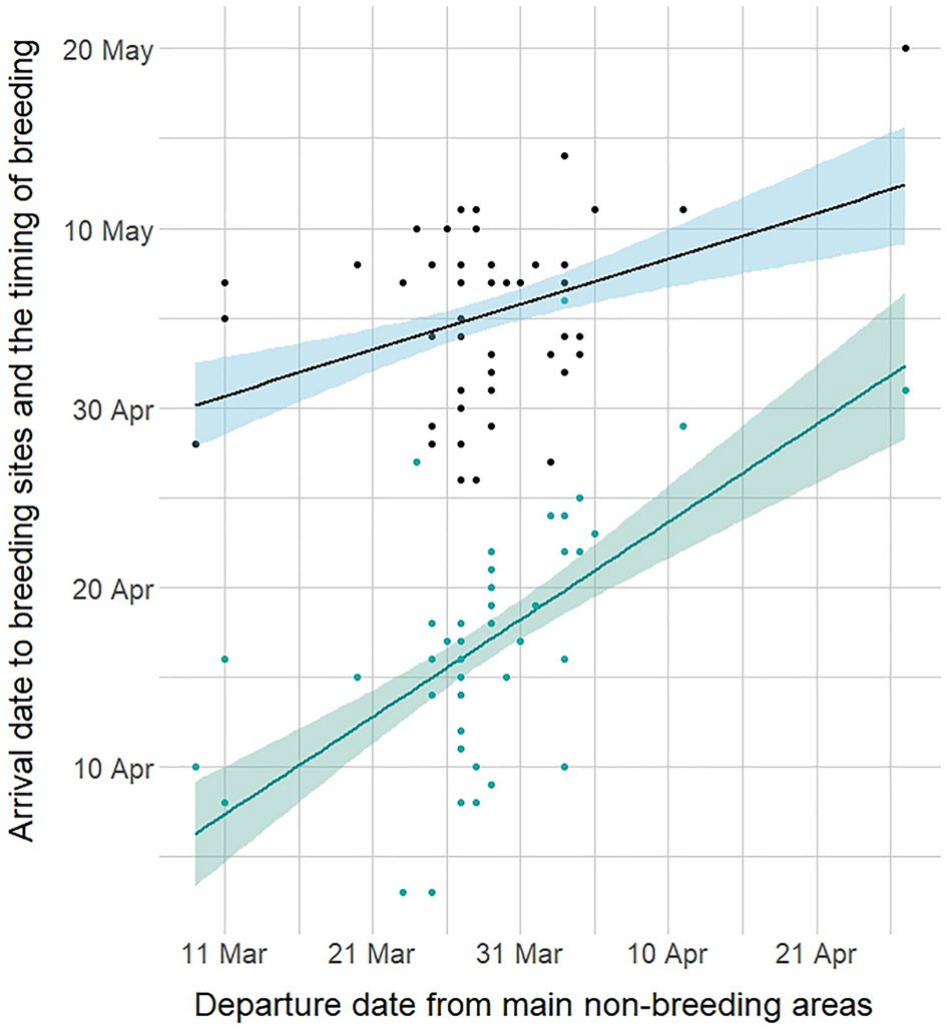
Positive relationships between arrival date to European breeding sites with the non-breeding departure date from African non-breeding grounds shown in green, and subsequently between the timing of breeding (egg laying date) with the non-breeding departure date from African non-breeding grounds shown in black, in pied flycatchers tracked with geolocators from both breeding populations combined. Shading indicates 90% confidence intervals.

Arrival to breeding sites was from 03 April to 08 May, average 17 April, and was linearly related to departure date from non-breeding areas (*n* = 56, Fig. 2, Table 2, model sets provided in Supplementary Table S4), and sex, with males arriving 8 days before females (average male arrival 14 April, female arrival 22 April). Early departing pied flycatchers had a longer duration migration but arrived to breeding sites earlier.

**Table 2:** Candidate models testing an association between date of departure from non-breeding areas and arrival to breeding sites.

(a) Component	Log likelihood	AICc	ΔAIC	Weight
Departure date + Sex	**− 168.03**	**344.8**	**0**	**0.57**

(b) Fixed effects	Estimate ± SE	95% CI	*t* value	*P* value
Departure from non-breeding areas	0.35 ± 0.09	0.16/0.53	3.80	<0.001
Females	− 6.28 ± 1.67	− 9.62/− 2.94	− 3.78	<0.001

The best supported model is shown in bold, and there were no other models within 4 ΔAIC of this top model (a). Parameter estimates, adjusted standard error (SE), 95% confidence intervals (CI), and significance values from the best supported model (b).

### Breeding phenology and fitness

On average the first egg was laid 06 May (mean of 19 days after arrival). Timing of breeding was related to departure date from non-breeding areas (Fig. 2) with earlier departing individuals associated with earlier egg laying, and individuals in the Netherlands laying earlier than those in the UK (average first egg date in the Netherlands 04 May compared to 08 May in the UK, *n* = 44, Table 3, model set are provided in Supplementary Table S5). These effects were independent of spring migration duration, which was weakly negatively associated with first egg laying date, and arrival date to breeding sites, which was weakly positively associated with first egg laying date (Supplementary Fig. S2).

**Table 3:** Candidate models testing an association between spring migratory timing (departure date from non-breeding areas and spring migration duration) and timing of egg laying.

(a) Component	Log likelihood	AICc	ΔAIC	Weight
Departure date + Population	**332.84**	**− 31.33**	**0.00**	**0.19**
Departure date + Population + Year	326.59	− 31.16	0.17	0.17
Departure date + Sex + Population	327.34	− 30.86	0.47	0.15
Departure date	345.27	− 29.61	1.71	0.08
Departure date + Sex + Population + Year	324.17	− 29.46	1.87	0.07
Departure date + Population	332.79	− 28.79	2.53	0.05
Departure date + Year	340.31	− 28.75	2.58	0.05
Departure date + Migration duration + Population + Year	326.54	− 28.49	2.84	0.05
Departure date + Migration duration + Sex + Population	326.71	− 28.42	2.90	0.04
Departure date + Sex	343.60	− 27.70	3.63	0.03
Departure date + Migration duration	344.69	− 27.36	3.96	0.03

(b) Fixed effects	Estimate ± SE	95% CI	*t* value	*P* value
Departure from non-breeding areas	0.37 ± 0.11	0.15/0.60	3.29	0.051
Population	− 3.90 ± 1.94	− 7.81/0.01	− 2.02	0.001

All models within 4 ΔAIC of the top model are shown, with the best supported model in bold (a). The log likelihood values are back transformed from the log scale. Parameter estimates, adjusted standard error (SE), 95% confidence intervals (CI), and significance values from the best supported model (b).

**Figure 3:**
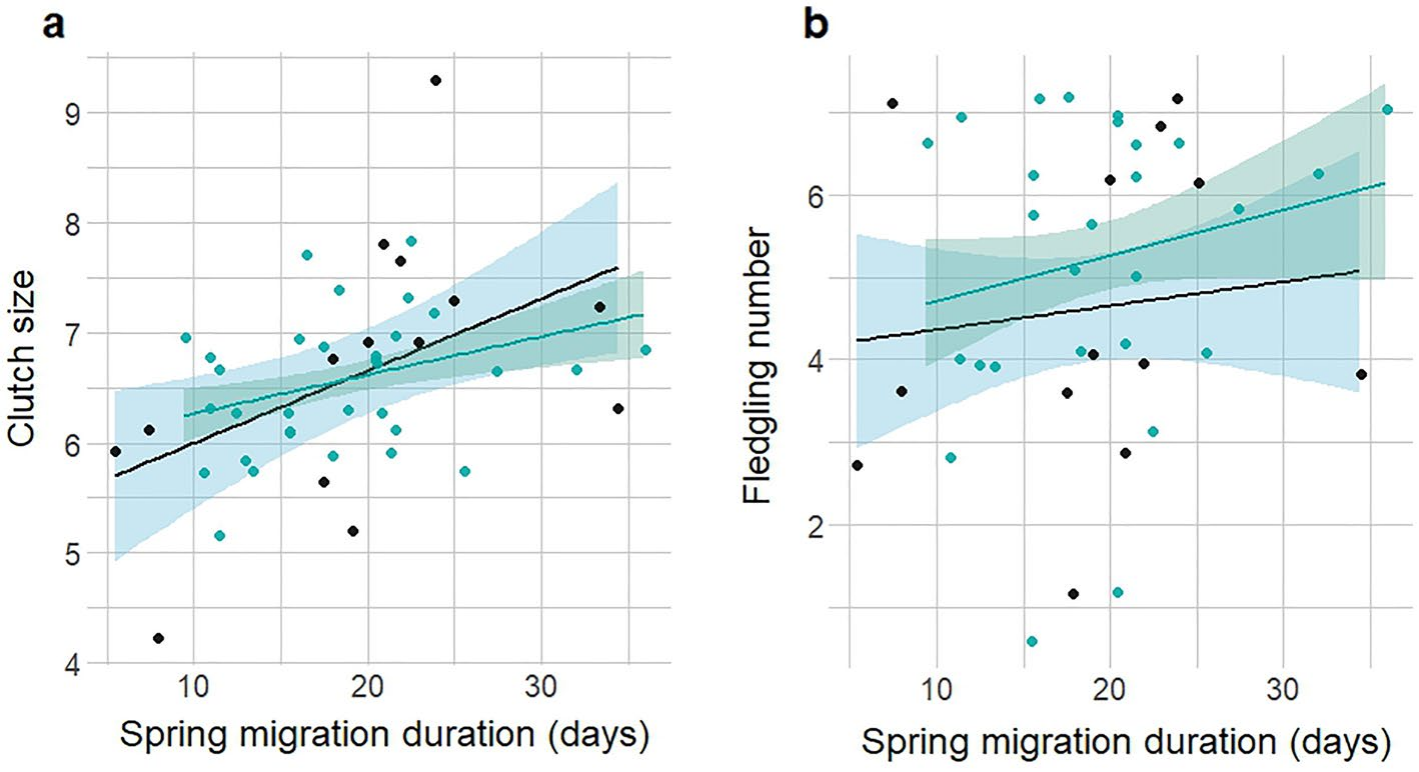
The positive relationship of spring migration duration on clutch size (**a**), and fledgling number (**b**) in geolocator tracked pied flycatchers, females are shown in black (14 & 13 respectively), males green (32 & 27 respectively). Duration is displayed in days, shading indicates 90% confidence intervals.

The relationship between measures of breeding performance and spring migration duration differed between sexes (Fig. 3, Table 4, model sets shown in Supplementary Table S6 and S7). Individuals that departed non-breeding areas earlier, and that had longer spring migration durations, produced a larger clutch size (n = 46, Fig. 3a). Spring migration duration was more important compared to departure date from non-breeding areas in this clutch size relationship (Supplementary Fig. S3), but arrival date at breeding sites had no effect on clutch size (Supplementary Table S6).

**Table 4:** Candidate models testing an association between duration of spring migration with clutch size (a) and (b), and fledgling number (c) and (d).

(a) Component	Log likelihood	AICc	ΔAIC	Weight
Migration duration	**− 55.45**	**117.47**	**0.00**	**0.34**
Migration duration + Population	− 55.19	119.35	1.88	0.13
Migration duration + Year	− 55.21	119.40	1.93	0.13
Migration duration + Sex	− 55.45	119.87	2.40	0.10
Migration duration + Population + Year	− 54.98	121.47	3.99	0.05

(b) Fixed effects	Estimate ± SE	95% CI	*t* value	*P* value
Migration duration	0.05 ± 0.02	0.01/0.08	2.745	0.009

(c) Component	Log Likelihood	AICc	ΔAIC	Weight
Migration duration*Sex + Clutch size	**− 77.61**	**169.61**	**0.00**	**0.32**
Migration duration*Sex + Clutch size + Population	− 77.12	171.53	1.91	0.12
Migration duration*Sex + Clutch size + Year	− 77.12	171.53	1.92	0.12
Clutch size	− 83.33	173.30	3.69	0.05
Migration duration*Sex + Clutch size + Population + Year	− 76.55	173.47	3.86	0.05

(d) Fixed effects	Estimate ± SE	95% CI	*t* value	*P* value
Migration duration * Sex	0.45 ± 0.14	0.16/0.75	3.15	0.003
Migration duration	− 0.32 ± 0.12	− 0.56/− 0.08	− 2.74	0.01
Sex	− 4.01 ± 1.58	− 7.21/− 0.82	− 2.54	0.02
Clutch size	0.87 ± 0.32	0.22/1.52	2.71	0.01

All models within 4 ΔAIC of the top model are shown, with the best supported model in bold (a) and (c). Parameter estimates, adjusted standard error (SE), 95% confidence intervals (CI), and significance values from the best supported model (b) and (d).

Accounting for clutch size, males with longer duration migrations fledged more young, yet the positive association between fledging success of females and migration duration was not important (*n* = 40, Fig. 3b). The effect of spring migration duration on fledgling number was independent of both departure date from non-breeding areas, and arrival date to breeding sites (Supplementary Table S7).

## Discussion

By tracking individual pied flycatchers across the spring migration and monitoring their subsequent breeding timing, clutch size, and fledgling number, we found that earlier departing and longer duration spring migration is associated with early arrival to breeding grounds for both sexes, and higher reproductive success for males. Departure from West African non-breeding areas was highly variable among individuals which was not explained by non-breeding longitude or the weather conditions we considered. We identified a strong influence of departure date on spring staging and migratory behaviour, and to subsequent timing of breeding. Individual departure phenology was maintained throughout both the spring migration and breeding period, with early departing individuals arriving at breeding areas and initiating breeding earlier, despite longer duration spring migrations, while later departing individuals arrived and initiated breeding later. A longer spring migration duration may provide a mechanism through which a carry-over effect can act, as longer spring migration duration was associated with a larger clutch size, and higher fledging success for males. It is possible our study overestimates such potential carry-over effects because archival geolocators only provide information for individuals that survive, and early timed individual may suffer higher mortality^45^.

Our study shows protandry throughout the spring migration, with males initiating migrations before females, as is common for long-distance migrant birds^28^. The extent of protandry was less on departure from West African non-breeding areas, with males departing a mean of 5 days before females, but males arrived by a mean of 8 days before females to breeding sites. This suggests that females tend to migrate for longer durations in spring, and stop more often or for longer, considering our tracked females stopped for a mean of 9 days, one day longer than males. This can be expected as female investment in breeding is greater and may necessitate increased time refuelling at staging areas^46^.

Departure date from West African non-breeding areas was variable among individuals, occurring over a 47-day period in March and April, with early individuals commencing breeding before the latest departing individuals had left West Africa. Ouwehand & Both^35^ found a 5-week variation in departure date from West Africa in an earlier study of the same Netherlands breeding population, however we likely found higher variation in departure date as our sample size was larger (67 compared to 14) and we tracked birds from four years and two breeding populations, compared to one. Such a large variation in departure date has been observed in other single-brooded migratory songbirds^25,47^. For pied flycatchers, West African location did not explain this variation in departure, neither did weather, contrary to our expectation that departure would correlate with weather conditions which assist migratory flight, such as a greater tailwind^20,48^. We included low altitude wind variables (at 10m height) to account for conditions experienced within non-breeding areas, but these variables are only available at a coarse scale due to data availability, and it is possible that weather variables at other altitudes or finer scales influence departure date.

Whilst environmental conditions may regulate broader departure phenology from West Africa^49^, the variability of departure in pied flycatchers could also be determined by genetic^50^ or physiological differences (such as mass or condition) among individuals^51^, including that related to local environmental variation or photoperiod^52^. An experimental food supplementation study conducted in Cote d'Ivoire increased the rate that pied flycatchers gained fuel stores pre-departure which resulted in greater variation in departure date, suggesting factors related to food availability are the main cues to departure, and those faster fuelling can advance departure^53^. The relative individual costs of an early compared to late departure date is little known; a late departure may increase risks of phenological mismatch with conditions or resources at spring stopover sites or at breeding grounds^10^, while migrating early can incur costs associated with increased risk of encountering unfavourable weather conditions or lower food availability^24^.

In our study individuals that departed earlier from non-breeding areas had longer duration spring migrations (Fig. 1a). Although within-species variation in route or flight speed could explain a small amount of duration variation, it mainly relates to the total duration of staging at stopover sites within Morocco or the Iberian Peninsula^37^, with earlier departing individuals stopping for longer compared to late departing individuals^54,55^ (Fig. 1b). The total spring migration was on average 19 days, similar to the 14 days found by Ouwehand & Both^35^ although the previous study did not find longer duration migrations for earlier departing individuals, which may be due to the smaller single year sample. The time spent at stopover sites, and hence migration duration, could be determined by pre-migration fuelling rate, with individuals departing earlier fuelling less pre-departure compared to later departing individuals, and instead gaining the resources needed to complete migrations at stopover sites enroute^56^. Such behaviour has been observed in Swainson’s thrushes *Catharus ustulatus*, where individuals using higher quality habitats departed later and stopped for less time during spring migration compared to those using lower quality habitats^25^, similarly late departing American redstarts *Setophaga ruticilla* compensated by increasing migration speed, but this had associated survival costs^26^. Resource availability and weather conditions at stopover sites may also influence stopover site staging duration^33^.

Increasing stopover duration may enable individuals to increase recovery and refuelling opportunity, acting as a buffer from adverse conditions experienced^57^. Sand storms over the Sahara Desert on spring migration can cause migration delays, reduce individual physiological condition and increase mortality^6,58^. As such, earlier departure may better enable individuals to flexibly adjust stopover duration, thereby increasing the likelihood of a successful migration^59^. This tactic may also enable optimisation of arrival date at breeding grounds such as reducing risks associated with arriving too early^24^. Temperature change and vegetation greening could be environmental departure cues for individuals from stopover sites^60^, allowing individuals that depart early to use spring stopover sites in North-west Africa and the Iberian Peninsula to better predict conditions at breeding sites than those with later phenologies^61^.

Despite having longer duration spring migrations, earlier departing individuals from West African non-breeding areas also arrived at European breeding sites earlier, with the order of departure maintained to arrival at breeding sites (Fig. 2). This general spring migration timing pattern is found in many other songbirds^62^, including in pied flycatchers from the same Netherlands breeding population^35^.

We identified a potential link between the timing of departure from non-breeding areas with subsequent timing of breeding, with early departing individuals also breeding earlier (Fig. 2). Early arrival generally enables benefits related to breeding, from securing a higher quality territory^63,64^, increased pairing opportunity, and a higher likelihood of matching breeding timing with seasonal food resources^39^. Early breeding is also commonly associated with positive fitness benefits in songbirds, including pied flycatchers, as early breeders tend to lay larger clutches and produce more fledglings and recruits^12,65^.

Our results suggest that pied flycatchers undertaking earlier and longer duration spring migrations have associated breeding benefits (Fig. 3), as these individuals were associated with larger clutches, and nests with early arriving males fledged more young once clutch size was taken into account. The latter effect was independent of departure phenology, suggesting a carry-over effect may arise from factors experienced on spring migration. Examples of mechanisms through which a carry-over effect may act include factors promoting longer staging^66^, more frequent stopping to avoid adverse weather conditions, optimisation of arrival time, or to await favourable weather for migratory flight similar to the use of temporary stopovers observed in larger birds^67,68^.

Our results indicate that fitness benefits associated with a longer spring migration may be positively carried over to influence reproductive success for both males and females, with migration duration a possible mechanism for this carry-over effect to act on, as in other migratory songbirds^14^. For females, a longer duration migration may ensure arrival in good pre-breeding condition, enabling the laying of larger clutches^11^. Similarly for males, arrival in good condition may improve reproductive output through an increased ability to secure higher quality territories and breed early^23^. Reproductive development of testes and ovaries starts during spring migration in pied flycatchers^69^, and so longer duration spring migration could result in arriving to breeding areas in an advanced reproductive condition and enable earlier breeding in response to suitable local environmental conditions.

An important caveat to our study is that the archival tracking devices we used only provide data for individuals that survive migrations. Early departing individuals may suffer higher mortality than later departing individuals during spring migration, and if so the exclusion of such information could result in an overestimation of the fitness benefits of early migration phenology. Studies tracking individual songbirds across annual cycles are currently only possible for small songbirds using archival dataloggers, although multi-sensor loggers are becoming available which will deepen our understanding through better interpretation of migration activity such as flight and stopover site use^70^. Multi-sensor loggers also enable more detailed insight into flight altitudes, migratory behaviours, and activity patterns, which will enhance understanding of migratory tactics, such as departure decisions. Linking these with higher resolution weather datasets will provide a better understanding of how weather interacts with migration behaviours. Ultimately however tags for small songbirds that provide information for individuals that do not survive as well as those that survive are required to fully answer these questions.

## Methods

### Ethics statement

All bird handling and geolocator fitting protocols were conducted according to the relevant national and institutional regulations on animal welfare, undertaken by licensed individuals, and UK protocols were carried out under licenses granted by the British Trust for Ornithology Special Marks Technical Panel. All bird handling and geolocator fitting protocols were approved by either the Netherlands Food and Consumer Product Safety Authority, or the University of Exeter Penryn Campus Ethics Committee, and are in accordance with ARRIVE guidelines.

### Study populations, breeding and migration data

Breeding pied flycatchers were monitored during 2016-2020 at two nest box populations, East Dartmoor in south-west England (50°36'N, 3°43'W), and Drenthe in the Netherlands (52°48'N, 6°24'E). East Dartmoor has a mean altitude of 200m and is dominated by deciduous oak and Drenthe is comprised of mixed coniferous and deciduous woodland at an altitude of 3 m.

Nest boxes were monitored at least twice weekly to determine first egg-laying date and complete clutch size. After 12 days of incubation nests were visited daily to check for hatching, until hatch date was determined. Visiting nests within 7 days after the expected fledging date identified the fledging number, defined as the number of young at the last pre-fledging inspection minus dead young left in the nest. For further details of East Dartmoor field methods see Bell et al.^37^ and Both et al.^71^ for details on Drenthe.

To study spring migration we fitted geolocators to adult pied flycatchers caught during breeding, females during incubation and males when holding a territory or provisioning nestlings. A total of 147 pied flycatchers at Dartmoor and 151 at Drenthe (Supplementary Table S1) were tagged over all years 2016-2019. There was a smaller female sample size due to sex specific caution of potential tag effects in earlier years of the project when tag models used were heavier, and female deployments were not licensed in the UK in 2018 to first make an assessment of return rates of 2017 tagged females.

Geolocators were fitted using a Rappole-Tipton leg loop harness^72^ made from 0.7mm elastane. Harness span was determined based on previous deployments on pied flycatchers, with several pre-assembled geolocators and harnesses available at deployment to reduce processing time. All geolocators were Migrate Technology Intigeo models which varied between population and year (P30Z11-7-DIP, P50Z11-7-DIP and W50Z11, Supplementary Table S1).

### Weather data

National Centre for Environmental Prediction data were obtained through the R reanalysis package RNCEP^73^, which accesses the National Oceanic and Atmospheric Administration (NOAA) database. We considered wind and temperature as the most likely direct cues of departure and extracted wind and temperature variables to estimate the conditions individuals encountered at the period of departure from West African non-breeding areas. Measures of air temperature (measured in kelvin at 2m height) and U and V wind (U component is positive for west to east wind, whilst V is for positive for south to north, with both measured in metres per second at 10m height) were downloaded in a gaussian grid format at a spatial resolution of 2.5° latitude and longitude, and a temporal resolution of 6 hours. Daily averages for each weather variable were calculated, then spatiotemporally matched to individuals non-breeding location as identified by tracking analysis. Wind direction bearing° was calculated on the basis of U and V wind components, then categorised into either tailwind, headwind or sidewind. For northwards spring migration, we assumed that all wind between north north-west, and north north-east were headwind (< 22.5° or > 337.5°), wind between south south-east and south south-west were tailwind (157.5°–202.5°), and other winds were sidewind.

### Geolocation analysis

We used the threshold method to derive location estimates from recorded light data^74^. In short, light values were initially log-transformed and sunrise and sunset times were defined using the R-package TwGeos^75^.

Movement and stationary periods (> 2 days) were defined based on changes in sunrise and sunset times using the R-package GeoLight v2.0.1^74,76^. To derive reference zenith angle for location estimates a tag specific Hill-Eekstrom calibration was performed for the longest stationary period during the non-breeding season^74,77^. With the derived zenith angle, a spatial mask allowing stationary location estimates on land only, and a movement model restricting the maximum distance of movements between twilight times, we applied the group threshold model in the R Package SGAT^78^. The tuned Monte Carlo Markov Chain (MCMC) model was run for 2000 iterations to ensure convergence and allow calculation of the most likely track (median across MCMC chains) and credibility intervals (e.g., 95 percentile of the MCMC chains). Detailed description of the geolocation analysis is provided in the supplementary material.

Interpretation of the geolocation analysis focused on identifying the phenology, location, and behaviour of individuals during the entire spring migration, from the period around departure from non-breeding areas to arrival at breeding sites. This included the estimated location of individuals during the non-breeding period, departure date from these areas, the use and location of staging areas (arrival to, departure from and duration of staging event) and arrival to breeding sites. Spring migration duration was calculated as the total duration between departure from the main non-breeding site in West Africa, to arrival at the European breeding site. One female with a spring migration duration of 58.5 days was excluded from all analysis as we considered this duration potentially erroneous being 39.3 days longer than the average spring migration duration of 19.2 days, and therefore possibly an artefact of variable sensor shading.

### Statistical analysis

#### Departure date from non-breeding area

Time-dependent Cox proportional hazard models (time-to-event analysis) were used to assess the effect of weather on the night-to-night probability of departing from non-breeding areas (R package survival^79^). These models describe the probability of the departure event occurring over time as a function of a hazard, here time-dependent weather conditions. Unlike other methods, Cox proportional hazard models account for conditions on nights prior to departure, when an individual did not depart, in addition to the night of departure providing greater insight into departure decisions^48^.

Daily weather estimates in the 30 days prior to departure were used^80^. We estimated the departure probability as a function of time-dependent weather variables (air temperature, wind speed and direction) matched to an individual’s non-breeding location, the interaction between wind speed and direction (to provide a measure of relative sidewind, headwind and tailwind), and accounted for fixed effects of year and sex, breeding population (UK or Netherlands) and longitude of non-breeding location in West Africa. Air temperature and wind speed were included as numeric variables, but wind direction was a 3-level categorical variable within analysis.

#### Spring migration duration, arrival, and breeding phenology

General linear models (GLMs) with Gaussian errors were used in all subsequent analysis and included a year and population term (4-level and 2-level factors respectively) as fixed effects to account for potential differences between years or the two populations. In all models, date variables were included as Ordinal dates with 1 January set to 0 at the start of each year and included as a numeric parameter.

We selected best fitting models using ΔAICc criterion (< 4) using the *dredge* function within the R package MuMIn^81^, with model fit assessed using pseudo r^2^ calculated using the *r.squareGLMM* function within the same package. Model validation was performed in the DHARMa package^82^ and variables checked for collinearity in the car package^83^. Corrected Akaike’s Information Criterion (AICc), change in AICc (ΔAICc) and model weight calculated using the *dredge* function indicate support for candidate models. Spring migration duration was defined as the total number of days between departure from non-breeding areas and arrival to breeding sites.

Spring migration duration was modelled as the response variable in relation to non-breeding area departure date, with additional categorical fixed effects of year, population, and sex. Arrival date to breeding sites was modelled as the response variable in relation to non-breeding area departure date and categorical factors of year, population, and sex.

Two GLMs investigated how breeding phenology was influenced by spring migratory tactic. Egg laying date was the response variable in both models, which was log transformed to improve data fit to a normal distribution. As measures of migratory timing that were strongly correlated, departure from non-breeding areas and spring migration duration were included in one model as fixed effects (departure from non-breeding areas VIF score 2.3, spring migration duration VIF score 1.9), while arrival to breeding sites was included as an explanatory factor within a second model (arrival date was modelled separately due to multicollinearity when included alongside other measures of migratory timing, VIF score >100). Categorical factors of year, population and sex were explanatory factors within both models.

Two sets of GLMs tested how breeding factors were influenced by spring migratory tactic, one set with clutch size as the response variable, another with fledgling number. Three models were run for the clutch size analysis, each including an interaction term between sex and a single explanatory measure of migratory timing; either departure from non-breeding areas, spring migration duration, or arrival at breeding sites. These measures of migratory timing were modelled separately due to multicollinearity when more than one was included (VIF score >100 in all instances). Categorical factors of year and population were additional explanatory factors and data that related to 6 males which failed to pair were omitted. A further three models were run for the fledgling number analysis, with the same model structure used for clutch size but with the addition of clutch size as an explanatory variable to account for any environmental effects influencing clutch size and hence number of fledglings, excluding data relating to 6 individuals that failed during breeding (to account for environmental or predation related events).

### Supplementary Information

Supplementary Information.

## Data Availability

The datasets generated during and/or analysed during the current study that relate to UK breeding birds are available in the Movebank Data Repository, 10.5441/001/1.1732qn7j. To request the Netherlands breeding data contact Christiaan Both c.both@rug.nl and contact Malcolm Burgess malcolm.burgess@rspb.org.uk to request the UK breeding data.
