# Supplementary material for: Individuals departing non-breeding areas early achieve earlier breeding and higher breeding success
Source: Sci Rep. 2024 Feb 19;14:4075. doi: 10.1038/s41598-024-53575-2 (PMC10876959; doi:10.1038/s41598-024-53575-2)
Supplement: Supplementary file 1 — Supplementary Information. [file 41598_2024_53575_MOESM1_ESM.pdf]

Individuals departing non-breeding areas early achieve earlier breeding and higher breeding success

Fraser Bell[1,2*], Janne Ouwehand[3], Christiaan Both[3], Martins Briedis[4,5], Simeon Lisovski[6], Xuelai Wang[3], Stuart Bearhop[1], Malcolm Burgess[2,7,8]

[1]Centre for Ecology and Conservation, University of Exeter, Penryn, Cornwall, UK

[2]Royal Society for the Protection of Birds, Centre for Conservation Science, The Lodge, Sandy, Bedfordshire, UK

[3]Conservation Ecology Group, University of Groningen, Groningen, The Netherlands

[4]Department of Bird Migration, Swiss Ornithological Institute, Sempach, Switzerland

[5]Lab of Ornithology, Institute of Biology, University of Latvia, Rīga, Latvia

[6]Alfred Wegener Institute for Polar and Marine Research, Telegrafenberg, Potsdam, Germany

[7]Centre for Research in Animal Behaviour, University of Exeter, Exeter, Devon, UK

[8]PiedFly.Net, Yarner Wood, Bovey Tracey, Devon, UK

*** Corresponding author contact address:** fraserbell@hotmail.co.uk or fraser.bell@rspb.org.uk

Supplementary information

Supplementary Table 1. Summary of geolocator deployments. Body mass was based on the average mass of all adult pied flycatchers measured across the study sites (12.72g). Some tags could not be analysed due to tag or battery failure (see Supplementary Figure 2).

Study population	Year	Model	Light pipe	Tag weight (mean % load of body mass)	Total deployed			Total retrieved and percentage return rate			Tags not analysed
					Total	Male	Female	Total	Male	Female	
Dartmoor	2017/18	P30	Yes	0.42 (3.3%)	79	42	37	19	13 (31%)	6 (16%)	1
	2018/19				22	22	-	11	11 (44%)	-	2
	2019/20				46	26	20	14	7 (27%)	7 (35%)	3
Drenthe	2016/17	W50	No	0.50 (3.9%)	20	20	-	6	6 (30%)	-	1
		P50	Yes	0.51 (4.0%)	21	21	-	4	4 (19%)	-	-
	2017/2018	W50	No	0.50 (3.9%)	28	28	-	5	5 (18%)	-	2
		P50	Yes	0.51 (4.0%)	12	12	-	2	2 (17%)	-	-
	2018/19	P30	Yes	0.42 (3.3%)	40	26	14	14	10 (71%)	4 (29%)	-
	2019/20				30	21	9	7	5 (56%)	2 (22%)	1

Detailed description of the geolocation analysis

We used the threshold method to derive location estimates from the recorded light data[1]. First, we log-transformed light values and defined sunrise and sunset times in the R-package TwGeos[2] using a default light intensity threshold of 0.8 log-lux and the *preprocessLight* function. For a single tag a slightly higher threshold of 1 lux was applied as a slight upward shift in baseline values was observed. The resulting sun-events were cross-referenced with the automated *twilightEdit* function, to check if large outliers (of more than 45 minutes) were excluded within subsequent analysis.

We distinguished between movement and stationary periods applying the *invChanges* and *extractMovements* function from the R-package GeoLight v2.0.1[3]. Based on this, twilight events were grouped in either movement or stationary periods; a default minimum duration of each period

were set to 4 twilight events, which corresponds to 2 days (in 16% of instances minimum duration was set to 3 events or 1.5 days, to allow for identification of distinct short stops). Due to variation in the levels of ambient shading and the extent of movement unique to individuals (for example different breeding origin or migratory routes), we used tag specific settings for the thresholds in both the *extractMovements* function (for sunset, sunrise, noon, midnight; values used averaged at 0.7, IQR 0.68 - 0.76 and range 0.4 - 0.85) and *mergeGroups* function (that defines how much proportion of overlap is needed to merge two consecutive periods). The minimum value set to merge periods which overlapped by 63%, and the maximum 95% (average 80%).

We modelled individual migration tracks and stationary periods using the R-package SGAT[4] which applies a Bayesian framework combining the observed sunrise and sunset data with a priori knowledge of bird behaviour to provide location estimates with the associated uncertainty[1]. To this end, we used the group threshold model with the predefined stationary and movement periods described above and allowed the birds to move in any direction but defined that stationary areas must be located on land. The group threshold model assumes the error in twilight times follows a gamma distribution which will be representative across the entire year.

For estimating a representative shape of the gamma distribution and zenith0 angle (referring to the least shaded conditions) of light errors, for each unique tag, we attempted an in-habitat calibration for each tag but it failed in many cases (24 tags), most likely because of staging and moult. As such, models underpinned with in-habitat SEA calibration appeared to estimate results which were un-representative across the whole annual cycle, indicating the calibration period was inadequate for periods outside the calibration period. To overcome this, we used a tag-specific zenith0 (a median zenith0 from 10 sampling iterations per tag) from a gamma light error distribution that was inferred using a Hill-Ekstrom sampling procedure that seeks to find zenith angles where variance in latitude positions is minimized. The longest defined stationary period was during the non-breeding season[5,6]. This period is likely to be less influenced by shading and be more representative of for the non-breeding period as a whole (than when using the breeding period),

which is vital for studies focussed on identifying behaviours during the migration or non-breeding period. Code and further details on the Hill-Ekstrom calibration to derive zenith angles and parameters for the log-normal twilight distribution are provided in Lisovski[7].

For each tag, the final group threshold models were parameterised using a twilight model with the zenith0 shape and scale parameters set to 2.5 and 0.1 respectively. Such broad error distribution was used to reduce the confidence that the resultant peak zenith is representative for all stationary non-breeding periods. The flight speed probability distribution was assumed to follow a gamma distribution (shape = 2, scale = 0.1), implying relatively few fast movements which would correspond to migration between the stopover sites. For the initial path, we extrapolated latitudinal positions during the equinox periods by setting the tolerance of solar declination of up to 8°. We initiated the model by drawing 1000 initial samples from a *modifiedGamma* model with relaxed assumptions and then tuned the model five times with all assumptions/priors implemented (Gamma model; 300 iterations each). The model was run for 2000 iterations to ensure convergence. From the resulting 2000 chains, we extracted median location estimates and their 95% credibility intervals of each stationary period and of each twilight during the movement. Models failed to run in 6 instances as batteries failed during the November – February period and tags could not be calibrated to the core non-breeding period.

Supplementary Figure 1. Visual summary of data and sample sizes

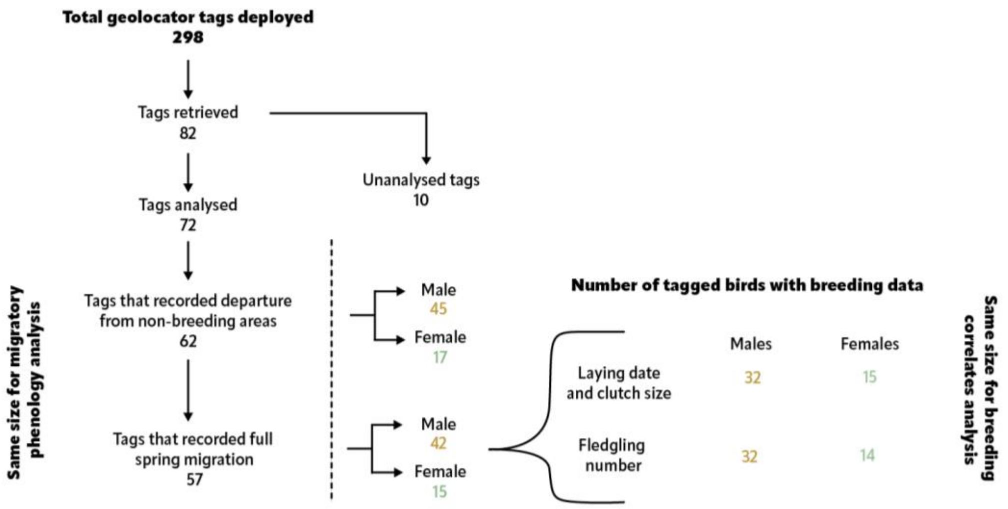

Supplementary Table 2. Effects of weather variables, year, sex, and population on the probability of departure from non-breeding areas in West Africa (62 events), within a 30-day weather window prior to the date of departure **a)**. The same model without the inclusion of year to account for possible between-year variation **b)**.

Parameter	Hazard Ratio	95% CI	p-value
**a) Full model**			
Headwind	0.45	0.01, 23.2	0.7
Tailwind	0.52	0.05, 5.84	0.6
Wind speed	1.03	0.74, 1.43	0.9
Air temperature	1.10	0.96, 1.27	0.2
Longitude in West Africa	1.06	0.81, 1.37	0.7
Breeding in the Netherlands	0.57	0.21, 1.56	0.3
Year	0.92	0.67, 1.26	0.6
Males	1.55	0.79, 3.03	0.2
Headwind * wind speed	1.39	0.39, 5.02	0.6
Tailwind * wind speed	1.17	0.41, 3.40	0.8
**b) Reduced model without year**			
Headwind	0.47	0.01, 24.64	0.7
Tailwind	0.51	0.05, 5.67	0.6
Wind speed	1.05	0.77, 1.45	0.7
Air temperature	1.10	0.96, 1.26	0.2
Longitude in West Africa	1.06	0.82, 1.38	0.7

Breeding in the Netherlands	0.57	0.21, 1.57	0.3
Males	1.54	0.79, 2.99	0.2
Headwind * wind speed	1.36	0.38, 4.91	0.6
Tailwind * wind speed	1.20	0.42, 3.44	0.7

Hazard Ratio, 95% confidence intervals (CIs) and P-values are given. The UK population, female individuals, sidewind and the interaction between sidewind and wind speed are the reference parameters within the model.

Supplementary Table 3. Model sets that examine associations between the date of departure from non-breeding areas on spring migration duration. Models are ranked by AICc weight, with all models with weight >0.01 displayed. Degrees of freedom (df), second-order Akaike's information criterion values (AICc), delta AIC (ΔAIC), weight and pseudo R^2 are shown for all models. The most parsimonious model is indicated in bold.

Parameter	Intercept	df	AICc	ΔAIC	weight	R^2
**Departure date + Sex**	**80.7**	**4**	**344.3**	**0**	**0.73**	**0.50**
Departure date + Sex + Population	80.39	5	346.6	2.29	0.23	0.50
Departure date + Sex + Year	78.3	7	351.3	6.94	0.02	0.50

Supplementary Table 4. Model sets that examine associations between spring migratory timing on arrival date at breeding sites. Models are ranked by AICc weight, with all models with weight >0.01 displayed. Degrees of freedom (df), second-order Akaike's information criterion values (AICc), delta AIC (ΔAIC), model weight and pseudo R^2 are shown for all models. The most parsimonious model is indicated in bold.

Parameter	Intercept	df	AICc	ΔAIC	weight	R^2
**Departure date + Sex**	**81.02**	**4**	**344.8**	**0.00**	**0.57**	**0.46**
Departure date + Sex + Year	-487.1	5	347.1	2.26	0.18	0.47
Departure date + Sex + Population	80.73	5	347.2	2.31	0.18	0.47
Departure date + Sex + Population + Year	-506.7	6	349.5	4.65	0.06	0.47

Supplementary Table 5. Model sets that examine associations between spring migratory timing on egg laying date. Departure date from non-breeding areas and duration of spring migration were included in the first model i), and arrival at the breeding sites in the second ii). Models are ranked by AICc weight, with all models with weight >0.01displayed. Degrees of freedom (df), second order Akaike's information criterion values (AICc), delta AIC (ΔAIC), weight and pseudo R^2 are shown for all models. The most parsimonious models are indicated in bold.

Parameter	Intercept	df	**AICc**	**ΔAIC**	**weight**	**R^2**
**i) Departure date from non-breeding areas and spring migration duration**						
Departure date + Population	**2.72**	**4**	**-31.33**	**0.00**	**0.19**	**0.28**
Departure date + Population + Year	74.15	5	-31.16	0.17	0.17	0.32
Departure date + Sex + Population	2.48	5	-30.86	0.47	0.15	0.31
Departure date	2.71	3	-29.61	1.71	0.08	0.21
Departure date + Sex + Population + Year	57.45	6	-29.46	1.87	0.07	0.33
Departure date + Population	2.76	5	-28.79	2.53	0.05	0.28
Departure date + Year	63.21	4	-28.75	2.58	0.05	0.23
Departure date + Migration duration + Population + Year	75.20	6	-28.49	2.84	0.05	0.32
Departure date + Migration duration + Sex + Population	2.30	6	-28.42	2.90	0.04	0.31
Departure date + Sex	2.58	4	-27.70	3.63	0.03	0.22
Departure date + Migration duration	2.60	4	-27.36	3.96	0.03	0.21
Departure date + Migration duration + Sex + Population + Year	58.30	7	-26.92	4.40	0.02	0.34
Departure date + Migration duration + Year	69.62	5	-26.67	4.66	0.02	0.24
Departure date + Sex + Year	57.72	5	-26.28	5.04	0.02	0.24
Departure date + Migration duration + Sex	2.26	5	-25.87	5.46	0.01	0.23
Departure date + Migration duration + Sex + Year	59.15	6	-24.41	6.92	0.01	0.25
**ii) Arrival at the breeding sites**						
Arrival date	2.78	3	-23.71	0.00	0.16	0.09
Arrival date + Year	80.62	4	-23.49	0.21	0.14	0.14
Arrival date + Population + Year	86.17	5	-23.16	0.55	0.12	0.18
Arrival date + Population	2.91	4	-23.07	0.63	0.11	0.13
Population	3.64	3	-22.19	1.52	0.07	0.06
Null	**3.60**	**2**	**-21.75**	**1.95**	**0.06**	**0.00**
Arrival date + Sex	2.61	4	-21.54	2.16	0.05	0.10
Population + Year	69.49	4	-21.31	2.39	0.05	0.09
Arrival date + Sex + Population	2.67	5	-21.08	2.63	0.04	0.14

Arrival date + Sex + Year	79.08	5	-20.95	2.76	0.04	0.14
Arrival date + Sex + Population + Year	81.34	6	-20.57	3.14	0.03	0.18
Year	59.54	3	-20.50	3.21	0.03	0.02
Sex	3.63	3	-20.11	3.60	0.03	0.01
Sex + Population	3.65	4	-19.96	3.75	0.02	0.06
Sex + Year	82.93	4	-19.61	4.10	0.02	0.06
Sex + Population + Year	85.17	5	-19.56	4.14	0.02	0.11

Supplementary Figure 2. Relationships between date of first egg laying with the duration of spring migration (days) **i)**, and the arrival date to breeding sites **ii)**, for tracked pied flycatchers. Shading indicates 75% confidence intervals.

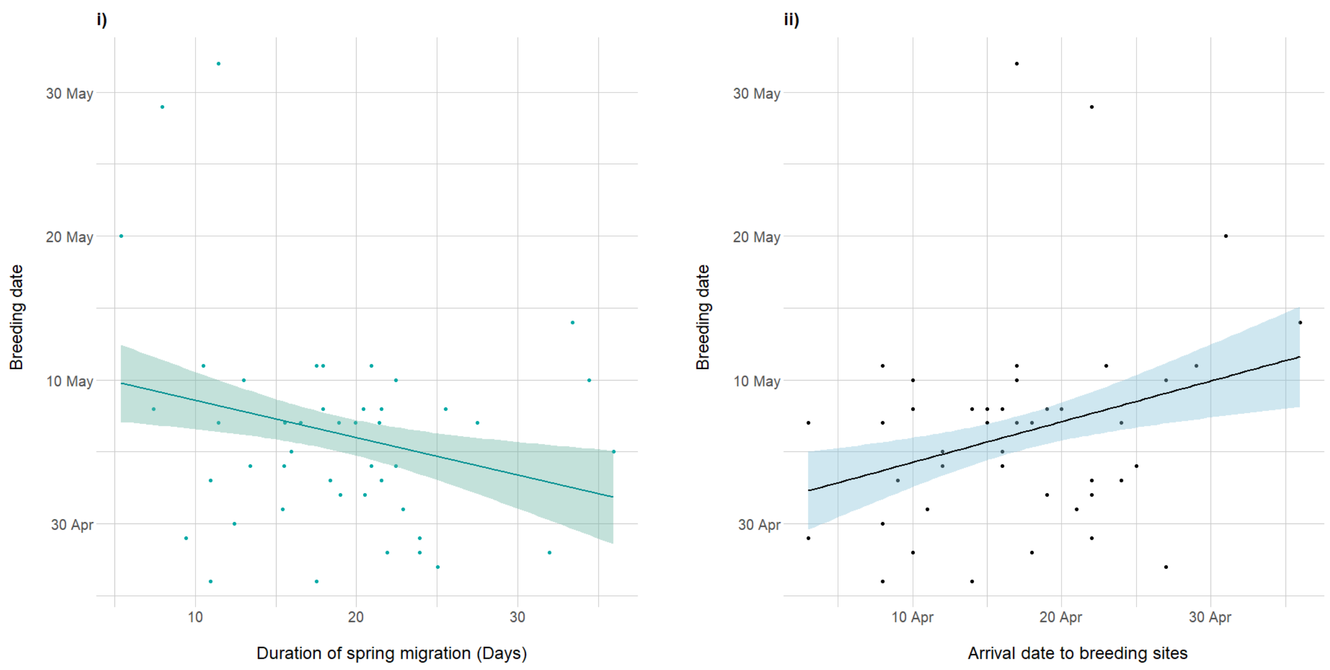

Supplementary Table 6. Model sets that examine associations between clutch size, and the interaction between sex and departure date from non-breeding areas i), duration of spring migration ii), and arrival at the breeding sites iii). Models are ranked by AICc weight, with all models with weight >0.01 displayed. Degrees of freedom (df), second order Akaike's information criterion values (AICc), delta AIC (ΔAIC), weight and pseudo R^2 are shown for all models. The most parsimonious models are indicated in bold.

Parameter	Intercept	df	**AICc**	**ΔAIC**	**weight**	**R^2**

**i) Departure date from non-breeding areas**						
Departure date + Sex	11.46	4	118.38	0.00	0.19	0.17
Departure date	10.00	3	118.73	0.35	0.16	0.12
Departure date + Population	10.05	4	119.67	1.30	0.10	0.15
Departure date + Year	-278.46	4	119.75	1.37	0.09	0.15
Departure date * Sex	13.33	5	119.88	1.50	0.09	0.19
Departure date + Sex + Population	11.28	5	120.30	1.92	0.07	0.18
Departure date + Sex + Year	-149.37	5	120.51	2.14	0.06	0.18
Departure date + Population + Year	-249.55	5	121.06	2.68	0.05	0.17
Departure date * Sex + Population	13.83	6	121.08	2.70	0.05	0.22
Departure date * Sex + Year	-121.62	6	122.26	3.88	0.03	0.20
Null	6.59	2	122.45	4.07	0.02	0.00
Departure date + Sex + Population + Year	-149.82	6	122.56	4.18	0.02	0.19
Population	6.71	3	123.20	4.82	0.02	0.03
Departure date * Sex + Population + Year	-109.95	7	123.63	5.26	0.01	0.22
Year	-263.15	3	123.68	5.31	0.01	0.02
Sex	6.64	3	124.66	6.28	0.01	0.00
Population + Year	-231.24	4	124.76	6.39	0.01	0.05
Sex + Population	6.71	4	125.60	7.23	0.01	0.03
**ii) Spring migration duration**						
Migration duration	5.67	3	117.47	0.00	0.34	0.15
Migration duration + Population	5.80	4	119.35	1.88	0.13	0.16
Migration duration +Year	-163.31	4	119.40	1.93	0.13	0.15
Migration duration + Sex	5.69	4	119.87	2.40	0.10	0.15
Migration duration + Population + Year	-150.74	5	121.47	3.99	0.05	0.16
Migration duration * Sex	5.35	5	121.61	4.14	0.04	0.16
Migration duration + Sex + Population	5.78	5	121.86	4.39	0.04	0.16
Migration duration + Sex + Year	-179.28	5	121.89	4.42	0.04	0.16
Null	6.59	2	122.45	4.98	0.03	0.00
Population	6.71	3	123.20	5.73	0.02	0.03
Migration duration * Sex + Population	5.38	6	123.28	5.80	0.02	0.18
Year	-263.15	3	123.68	6.21	0.02	0.02
Migration duration + Sex + Population + Year	-179.46	6	124.00	6.53	0.01	0.17
Migration duration * Sex + Year	-121.47	6	124.05	6.58	0.01	0.16
Sex	6.64	3	124.66	7.19	0.01	0.00
Population + Year	-231.24	4	124.76	7.29	0.01	0.05
Sex + Population	6.71	4	125.60	8.13	0.01	0.03
**iii) Breeding arrival date**						
Null	6.59	2	122.45	0.00	0.22	0.00

Arrival date	6.71	3	123.20	0.75	0.15	0.03
Year	-263.15	3	123.68	1.23	0.12	0.02
Population	6.64	3	124.66	2.21	0.07	0.00
Sex	6.78	3	124.73	2.28	0.07	0.00
Arrival date + Year	-231.24	4	124.76	2.31	0.07	0.05
Arrival date + Sex	7.32	4	125.51	3.06	0.05	0.04
Arrival date + Population	6.71	4	125.60	3.15	0.05	0.03
Sex + Year	-277.02	4	126.00	3.55	0.04	0.02
Population + Year	-269.82	4	126.08	3.63	0.04	0.02
Sex + Population	7.51	4	126.95	4.50	0.02	0.00
Arrival date + Sex + Year	-251.50	5	127.06	4.61	0.02	0.06
Arrival date + Population + Year	-261.21	5	127.18	4.73	0.02	0.05
Arrival date + Sex + Population	7.66	5	127.99	5.54	0.01	0.04
Sex + Population + Year	-266.72	5	128.51	6.06	0.01	0.02
Arrival date * Sex + Population	7.31	5	129.47	7.02	0.01	0.00
Arrival date + Sex + Population + Year	-257.73	6	129.71	7.26	0.01	0.06

Supplementary Table 7. Model sets that examine associations between fledgling number, and the interaction between sex and departure date from non-breeding areas i), duration of spring migration ii), and arrival at the breeding sites iii). Models are ranked by AICc weight, with all models with weight >0.01 displayed. Degrees of freedom (df), second order Akaike's information criterion values (AICc), delta AIC (ΔAIC), weight and pseudo R^2 are shown for all models. The most parsimonious models are indicated in bold.

Parameter	Intercept	df	**AICc**	**ΔAIC**	**weight**	**R^2**
**i) Departure date from non-breeding areas**						
Clutch size	1.61	3	169.35	0.00	0.10	0.06
Departure date	9.26	3	169.48	0.13	0.10	0.06
Null	**4.98**	**2**	**169.52**	**0.17**	**0.09**	**0.00**
Sex + Clutch size	0.82	4	169.82	0.47	0.08	0.10
Sex	4.50	3	170.37	1.02	0.06	0.04
Departure date + Clutch size	5.55	4	170.75	1.40	0.05	0.08
Clutch size + Population	1.24	4	171.34	1.99	0.04	0.07
Departure date + Sex	8.20	4	171.36	2.01	0.04	0.07
Departure date + Population	9.30	4	171.59	2.24	0.03	0.06
Population	4.88	3	171.68	2.32	0.03	0.00
Year	-123.88	3	171.80	2.45	0.03	0.00
Clutch size + Year	-6.78	4	171.81	2.46	0.03	0.06
Departure date + Year	-70.02	4	171.92	2.57	0.03	0.06
Sex + Clutch size + Year	-407.93	5	171.97	2.62	0.03	0.11

Sex + Year	-513.51	4	172.14	2.79	0.03	0.05
Departure date + Sex + Clutch size	3.05	5	172.16	2.81	0.03	0.11
Sex + Clutch size + Population	0.67	5	172.30	2.95	0.02	0.11
Departure date + Clutch size + Population	5.29	5	172.80	3.45	0.02	0.10
Sex + Population	4.49	4	172.82	3.47	0.02	0.04
Departure date + Clutch size + Year	0.39	5	173.35	4.00	0.01	0.08
Departure date * Sex	6.21	5	173.47	4.12	0.01	0.08
Departure date + Sex + Year	-353.10	5	173.64	4.29	0.01	0.08
Departure date + Sex + Population	8.40	5	173.82	4.47	0.01	0.07
Clutch size + Population + Year	-81.46	5	173.92	4.57	0.01	0.07
Population + Year	-180.49	4	174.04	4.69	0.01	0.01
Departure date + Population + Year	-141.54	5	174.12	4.77	0.01	0.07
Departure date * Sex + Clutch size	0.70	6	174.28	4.93	0.01	0.12
Sex + Clutch size + Population + Year	-431.37	6	174.55	5.20	0.01	0.12
Departure date + Sex + Clutch size + Year	-356.44	6	174.58	5.23	0.01	0.12
Sex + Population + Year	-526.69	5	174.70	5.35	0.01	0.05
Departure date + Sex + Clutch size + Population	3.20	6	174.71	5.36	0.01	0.11
**ii) Spring migration duration**						
**Migration duration * Sex + Clutch size**	**2.24**	**6**	**169.61**	**0.00**	**0.32**	**0.29**
Migration duration * Sex + Clutch size + Population	1.91	7	171.53	1.91	0.12	0.30
Migration duration * Sex + Clutch size + Year	-556.37	7	171.53	1.92	0.12	0.30
Clutch size	1.48	3	173.30	3.69	0.05	0.06
Migration duration * Sex + Clutch size + Population + Year	-590.74	8	173.47	3.86	0.05	0.32
Null	5.02	2	173.76	4.14	0.04	0.00
Sex + Clutch size	0.80	4	174.37	4.76	0.03	0.09
Migration duration * Sex	6.82	5	174.48	4.87	0.03	0.15
Clutch size + Population	1.04	4	174.98	5.37	0.02	0.08
Sex	4.67	3	175.17	5.56	0.02	0.02
Migration duration + Clutch size	1.54	4	175.67	6.06	0.02	0.07
Population	4.88	3	175.69	6.08	0.02	0.01
Clutch size + Year	-39.37	4	175.74	6.13	0.01	0.06
Year	-166.68	3	175.99	6.38	0.01	0.00
Migration duration	4.89	3	176.05	6.44	0.01	0.00
Migration duration * Sex + Year	-570.69	6	176.35	6.73	0.01	0.16
Sex + Clutch size + Population	0.54	5	176.51	6.90	0.01	0.10
Sex + Clutch size + Year	-383.33	5	176.56	6.95	0.01	0.10
Migration duration * Sex + Population	6.68	6	176.77	7.16	0.01	0.16
Sex + Year	-492.60	4	177.00	7.39	0.01	0.04
Sex + Population	4.60	4	177.43	7.82	0.01	0.03
Clutch size + Population + Year	-126.65	5	177.52	7.90	0.01	0.08
Migration duration + Clutch size + Population	1.09	5	177.54	7.93	0.01	0.08
Migration duration + Sex	4.52	4	177.59	7.97	0.01	0.02

**iii) Arrival at the breeding sites**						
Sex + Clutch size	8.95	4	168.58	0.00	0.11	0.13
Clutch size	12.38	3	168.68	0.10	0.11	0.07
Sex	1.61	3	169.35	0.77	0.08	0.06
**Null**	**4.98**	**2**	**169.52**	**0.94**	**0.07**	**0.00**
Sex + Population	0.82	4	169.82	1.24	0.06	0.10
Population	4.50	3	170.37	1.79	0.05	0.04
Clutch size + Year	-444.63	4	170.52	1.94	0.04	0.09
Sex + Clutch size + Year	-320.62	5	170.84	2.26	0.04	0.14
Sex + Clutch size + Population	7.28	5	170.96	2.38	0.03	0.14
Arrival date + Sex + Clutch size	8.45	5	170.96	2.38	0.03	0.14
Clutch size + Population	11.53	4	171.08	2.50	0.03	0.08
Arrival date + Clutch size	12.23	4	171.10	2.52	0.03	0.08
Arrival date + Sex	1.24	4	171.34	2.76	0.03	0.07
Arrival date	4.88	3	171.68	3.10	0.02	0.00
Year	-123.88	3	171.80	3.23	0.02	0.00
Sex + Year	-6.78	4	171.81	3.23	0.02	0.06
Sex + Population + Year	-407.93	5	171.97	3.39	0.02	0.11
Population + Year	-513.51	4	172.14	3.56	0.02	0.05
Arrival date + Sex + Population	0.67	5	172.30	3.72	0.02	0.11
Arrival date * Sex + Clutch size + Population	13.27	6	172.64	4.06	0.01	0.16
Clutch size + Population + Year	-556.85	5	172.82	4.24	0.01	0.10
Arrival date + Population	4.49	4	172.82	4.24	0.01	0.04
Arrival date + Clutch size + Year	-479.91	5	173.00	4.42	0.01	0.09
Sex + Clutch size + Population + Year	-454.35	6	173.11	4.53	0.01	0.15
Arrival date + Sex + Clutch size + Year	-370.80	6	173.28	4.70	0.01	0.14
Arrival date + Sex + Clutch size + Population	7.16	6	173.57	4.99	0.01	0.14
Arrival date + Clutch size + Population	11.53	5	173.66	5.08	0.01	0.08
Arrival date + Sex + Year	-81.46	5	173.92	5.35	0.01	0.07
Arrival date + Year	-180.49	4	174.04	5.46	0.01	0.01
Arrival date * Sex + Clutch size + Population + Year	-699.94	6	174.25	5.68	0.01	0.12
Arrival date + Sex + Population + Year	-431.37	6	174.55	5.97	0.01	0.12
Arrival date + Population + Year	-526.69	5	174.70	6.12	0.01	0.05

Supplementary Figure 3. The relationship between departure date from non-breeding areas on clutch size in tracked pied flycatchers, females are shown in black, males green. Shading indicates 75% confidence intervals.

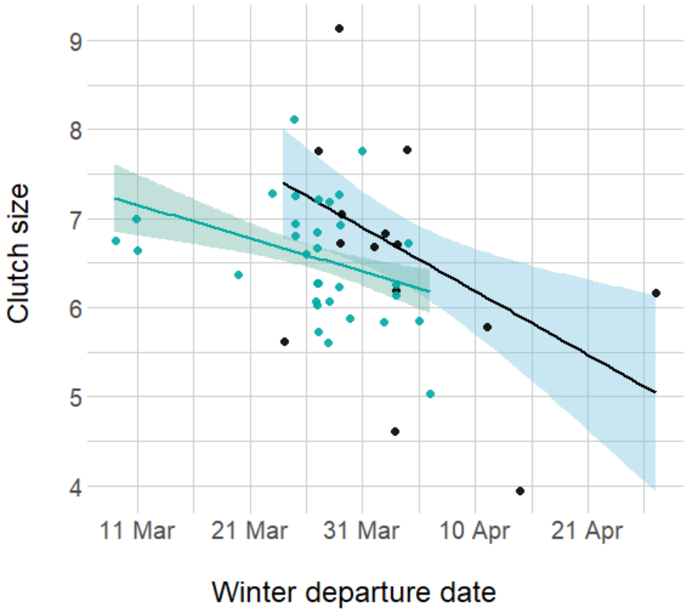

References

1. Lisovski, S., Bauer, S., Briedis, M., Davidson, S.C., Dhanjal-Adams, K.L., Hallworth, M.T., Karagicheva, J., Meier, C.M., Merkel, B., Ouwehand, J., Pedersen, L., Rakhimberdiev, E., Roberto-Charron, A., Seavy, N.E., Sumner, M.D., Taylor, C.M., Wotherspoon, S.J. & Bridge, E.S. (2020). Light-level geolocator analyses: A user's guide. *Journal of Animal Ecology,* **89**, 221– 236. **https://doi.org/10.1111/1365-2656.13036**

2. Lisovski, S., Sumner, M.D. & Wotherspoon, S.J. (2015). TwGeos: Basic data processing for light based geolocation archival tags. Github Repository. **https ://github.com/sliso vski/TwGeos**

3. Lisovski, S. & Hahn, S. (2012). Geo Light - processing and analysing light-based geolocator data in R. *Methods in Ecology and Evolution,* **3**(6), 1055– 1059. **https://doi.org/10.1111/j.2041-210x.2012.00248.x**

4. Wotherspoon, S.J., Sumner, M.D. & Lisovski, S. (2013). R package SGAT: solar/satellite geolocation for animal tracking. *GitHub Repository*. **https://github.com/SWotherspoon/SGAT**

5. Bell, F., Bearhop, S., Briedis, M., El Harouchi, M., Bell, S.C., Castello, J. & Burgess, M. (2022). Geolocators reveal variation and sex-specific differences in the migratory strategies of a long-distance migrant. *Ibis,* **164**, 451– 467. **https://doi.org/10.1111/ibi.13017**

6. Ouwehand, J., Ahola, M.P., Ausems, A.N.M.A., Bridge, E.S., Burgess, M., Hahn, S., Hewson, C.M., Klaassen, R.H.G., Laaksonen, T., Lampe, H.M., Velmala, W. & Both, C. (2016). Light-level geolocators reveal migratory connectivity in European populations of pied flycatchers *Ficedula hypoleuca*. *Journal of Avian Biology*, **47**, 69–83. **https://doi.org/10.1111/jav.00721**

7. Lisovski, S. 2023. SGAT Calibration Methods. Github Repository Release v0.0.1.
